# BCG instillation versus radical cystectomy for high-risk NMIBC with squamous/glandular histologic variants

**DOI:** 10.1038/s41598-019-51889-0

**Published:** 2019-10-24

**Authors:** Jungyo Suh, Kyung Chul Moon, Jae Hyun Jung, Junghoon Lee, Won Hoon Song, Yu Jin Kang, Chang Wook Jeong, Cheol Kwak, Hyeon Hoe Kim, Ja Hyeon Ku

**Affiliations:** 1 Department of Urology, Seoul Metropolitan Government-Seoul National University Boramae Medical Center, Seoul, South Korea; 2 Department of Urology Seoul National University College of Medicine, Seoul, South Korea; 3 Department of Pathology, Seoul National University Hospital, Seoul, South Korea; 4 Department of Urology, Pohang St Mary’s Hospital, PoHang, South Korea; 5 Department of Urology, Seoul National University Hospital, Seoul, South Korea

**Keywords:** Bladder cancer, Outcomes research

## Abstract

This study aims to evaluate the effect of Bacillus Calmette-Guérin (BCG) instillation and radical cystectomy on high-risk NMIBC with squamous or glandular variants. We retrospectively reviewed the data of high-risk (T1 or CIS or HG or TaG1/G2 with multiple, recurrent, large tumor) NMIBC patients from January 2000 to December 2017. Comparative analysis of radical cystectomy, intravesical BCG, and observation groups was conducted in high-risk NMIBC with squamous or glandular histologic variants. Among the 1263 high-risk NMIBC patient, 62 (4.9%) were reported squamous or glandular histologic variants. Thirty patients underwent BCG instillation and 15 patients were subjected to radical cystectomy. Statistically significant differences were found between the three treatment groups in terms of underlying hypertension (p = 0.031), T stage (p = 0.022) and tumor multiplicity (p = 0.019). Similar 5-year OS (p = 0.893) and CSS (p = 0.811) were observed in each of BCG instillation and radical cystectomy group. BCG instillation showed survival benefit in both OS (p = 0.019) and CSS (p = 0.038) than in the observation group. In high-risk patients diagnosed with NMIBC bladder cancer with squamous or glandular histologic variants, both intravesical BCG and radical cystectomy showed survival gain. In conclusion, BCG instillation represents an appropriate treatment option in high-risk NMIBC with squamous or glandular histologic variant.

## Introduction

Bladder cancer is the ninth most common malignancy and ranks thirteenth in terms of cancer-related deaths worldwide^1,2^. The most predominant histological phenotype is the urothelial carcinoma (UC), which constitutes about 90% of bladder cancer^3^. Muscle invasiveness is a critical landmark for the clinical evaluation of bladder cancer. Roughly 75% of patients present with non-muscle invasive bladder cancer (NMIBC)^4^ and most of them are easily treated with transurethral surgery. However, additional treatments such as early radical cystectomy or intravesical Bacillus Calmette-Guérin (BCG) instillation may be indicated in specific cases of NMIBC associated with a high risk of recurrence or progression.

Urothelial carcinoma shows potential pathological diversity resulting in concomitant histological variants. The incidence of histological variants in bladder urothelial carcinoma ranged between 10% and 25% in different studies^5^. Squamous and glandular differentiation, which are mostly coexistent^6,7^, are the most common subtypes followed by micropapillary, sarcomatoid and small cell types^8^. Most of the guidelines consider histological variants as high-risk disease^9^. Some of the variants, such as plasmacytoid or small cell types, show strong correlation with poor clinical outcome in NMIBC, underscoring the need for mandatory early radical cystectomy^10^. Micropapillary variants exhibit low response rates following intravesical therapy, warranting prompt radical cystectomy^11^. Squamous or glandular differentiation is related to poor response to chemotherapy in invasive muscle disease^12^; however, the clinical significance in NMIBC is still unclear.

BCG treatment is generally recommended for high-risk NMIBC, such as T1; high grade; concomitant CIS; multiple, recurrent, and large (>3 cm) Ta low-grade tumors. Early radical cystectomy is recommended for a few histologic variants, such as micropapillary or plasmacytoid types. However, its role in patients with the most common histologic variants including squamous and glandular manifestations, is unknown. In this study, we elucidated the clinical significance of BCG treatment for squamous and glandular variants of high-risk NMIBC.

## Material and Methods

### Ethics approval and consent

This study was approved by the Seoul National University Hospital (SNUH) Institutional review board (IRB) (No:H-1902-056-1009). As this was a retrospective study with anonymization of data, the IRB waived the requirement for informed consent from patients. All experiments were performed in accordance with relevant guidelines and regulations.

### Study population

**Figure 1:**
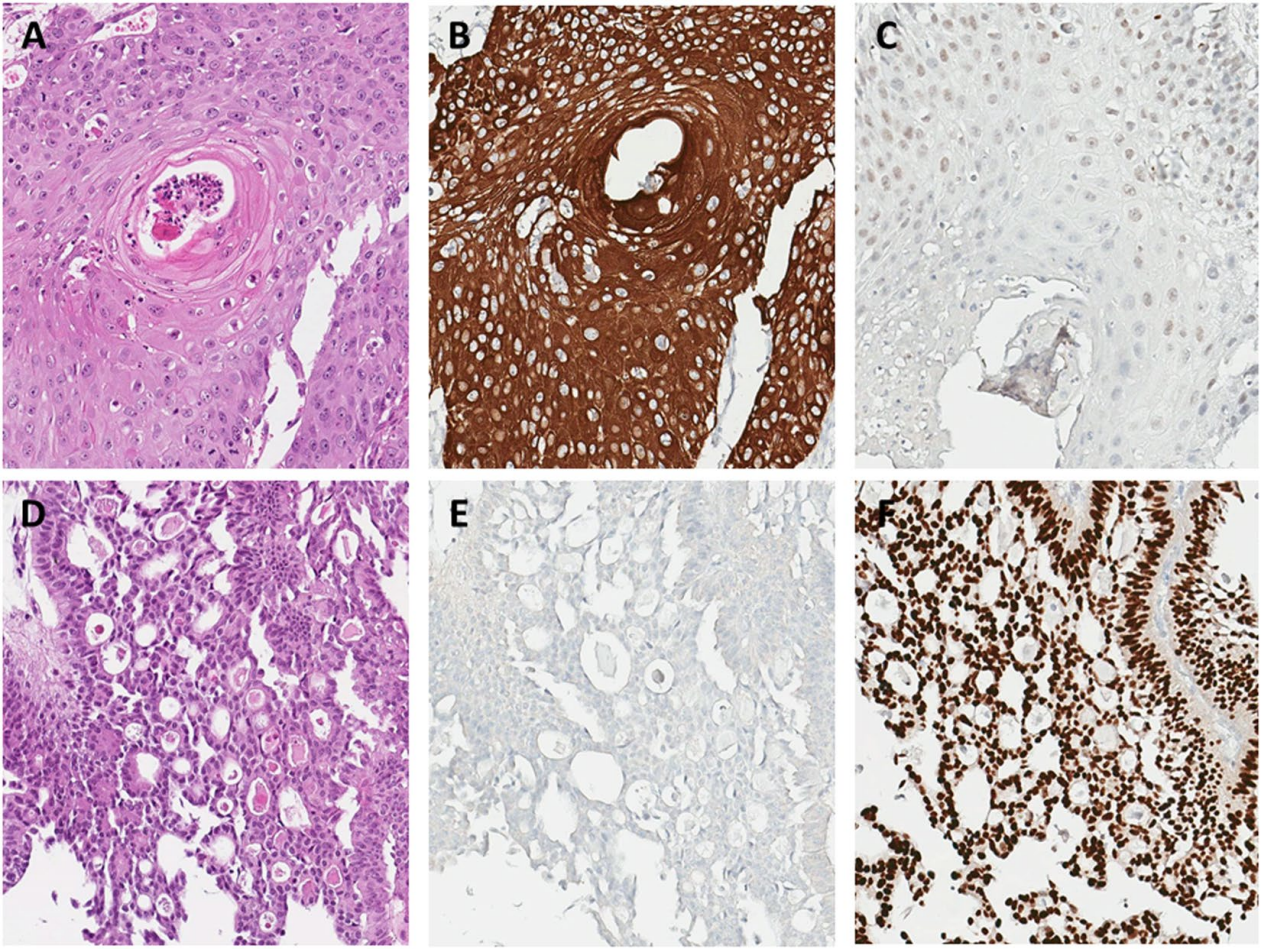
Typical histologic phenotype and immunohistochemistry staining status of squamous (**A**–**C**) or glandular (**D**–**F**) histologic variants of bladder cancer. Urothelial carcinoma with squamous differentiation (A) shows strong positive for CK14 (**B**) and weak to negative staining for GATA3. (**C**) Urothelial carcinoma with glandular differentiation (**D**) is negative for CK14 (**E**) and strong positive for GATA3 (**F**).

We retrospectively reviewed the electronic medical records of patients who underwent trans-urethral resection of bladder tumor (TURBT) and pathologically confirmed high-risk NMIBC between January 2000 and December 2017. High-risk NMIBC is defined by European Association of Urology (EAU) guideline as T1 tumor or carcinoma *in situ* (CIS) or high-grade (G3) or TaG1/G2 tumor with multiple, recurrent and large (>3 cm) cancer^13^. Among the 1263 high-risk NMIBC patients, 112 (8.8%) reported histologic variants in TURBT. Among the histologic variants, squamous or glandular subtypes were most commonly found in 62 patients (55.4%) followed by micropapillary (9.9%), plasmacytoid (1.8%) and sarcomatoid (1.8%) variants. Typical pathologic and immunohistochemistry features of urothelial carcinoma with squamous or glandular differentiation were demonstrated in Fig. 1.

Treatment methods included BCG instillation, radical cystectomy, and observation. Other instillation treatment methods were excluded for this analysis. Selection of BCG instillation or early radical cystectomy was followed by surgeon’s preference. None of them received adjuvant chemotherapy or radiotherapy. Observation group was consisted of the case of patient’s refusal for any treatment but agreed with regular follow up.

### Treatment and follow-up protocol

The BCG instillation protocol involved 6 cycles of weekly or biweekly induction and maintenance BCG in tolerant patients. The maintenance schedule included intensive instillation at three intervals: 3, 6, and 12 months in the first year, and every 6 months thereafter for 2 additional years. Radical cystectomy was performed with extended level lymph node dissection. None of the patients underwent radical cystectomy after BCG instillation.

Cystoscopy, urine cytology was performed for assessing recurrence of bladder cancer at every three months after the any of treatment cycle; including TURBT, induction and maintenance BCG. 1 week after 6 cycle of induction and every visit of follow-up. In the observation group, we checked cystoscopy and urine cytology by 3, 6, 12 months in first year and after that yearly followed up. Computed tomography (CT) scan was routinely performed in every 1-year follow-up. In the patients with gross hematuria or any symptoms that suspected recurrence, we performed CT scan for evaluating the disease status. Oncological outcomes were analyzed based on 5-year overall survival (OS) and cancer specific survival (CSS) in each of the treatment groups.

### Statistical analysis

Differences among the three treatment groups were tested by one-way ANOVA for continuous variables (age, and BMI) and chi-square test for categorial variables. Kaplan-Meier curves were calculated for OS and CSS with 95% confidence intervals (CIs). Log-rank test was used to compare the results of univariate analysis in each treatment group. All the statistical analyses were conducted using IBM SPSS Statistics 22. A p-value < 0.05 was considered significant.

## Results

### Patient characteristics

A total of 62 patients were analyzed in this study. Thirty patients underwent BCG instillation and 15 patients were treated with radical cystectomy. Underlying hypertension (p = 0.031), T stage (p = 0.022), and tumor multiplicity (p = 0.019) showed statistically significant differences between the three treatment groups. The patients selected for observation included those with a large proportion of low T stage (Ta, 35.3%) rather than BCG instillation (13.3%) and radical cystectomy (0.0%) groups. Detailed patient data are presented in Table 1.

**Table 1:** Characteristics of each treatment group. BMI; Body mass index, HTN; Hypertension, DM; Diabetes mellitus, GHU; Gross hematuria, CIS; Carcinoma *in situ*.

	Observation	BCG instillation	Radical cystectomy	P-value
Number	17	30	15	
Sex-male *– number* (%)	15 (88.2)	27 (90.0)	13 (86.7)	0.944^†^
Age (Years)	70.74 (±8.21)	67.60 (±9.93)	68.20 (±5.51)	0.564*
BMI (kg/m^2^)	29.68 (±9.9)	32.27 (±7.60)	24.00 (±6.36)	0.085*
HTN *– number* (%)	8 (47.1)	7 (23.3)	1 (6.7)	**0**.**031**^†^
DM *- number* (%)	4 (23.5)	5 (16.7)	0 (0.0)	0.152^†^
GHU history*- number* (%)	5 (29.4)	18 (60.0)	8 (53.3)	0.126^†^
T stage *– number* (%)				**0**.**022**^†^
Ta	6 (35.3)	4 (13.3)	0 (0.0)	
T1	11 (64.7)	26 (86.7)	15 (100.0)	
Tumor multiplicity *– number* (%)				**0**.**019**^†^
1	5 (29.4)	17 (56.7)	6 (40.0)	
2–7	6 (35.3)	12 (40.0)	3 (20.0)	
>8	6 (35.3)	1 (3.3)	6 (40.0)	
Tumor size >3 cm *– number* (%)	6 (35.3)	13 (43.3)	4 (26.7)	0.543^†^
Concomitant CIS *– number* (%)	2 (11.8)	6 (20.0)	3 (20.0)	0.751^†^

^*^Chi-square test, ^†^one-way ANOVA test.

Repeated TURBT was performed all of BCG instillation group, but only five (29.4%) in observation group. In early cystectomy group, all patient performed radical cystectomy without repeated TURBT. During the five years follow up periods, recurrence and progression was occurred in 10 patients (33.3%) and 4 patients (13.3%) in BCG treated group.

The patients with variant histology show significantly lower survival than pure urothelial carcinoma in both of OS (54.17 ± 0.54 months vs. 50.47 ± 1.87 months, p = 0.013) and CSS (55.41 ± 0.49 months vs. 52.78 ± 1.68 months, p = 0.038) in this cohort.

### Comparative oncological outcomes with different treatment modalities

**Figure 2:**
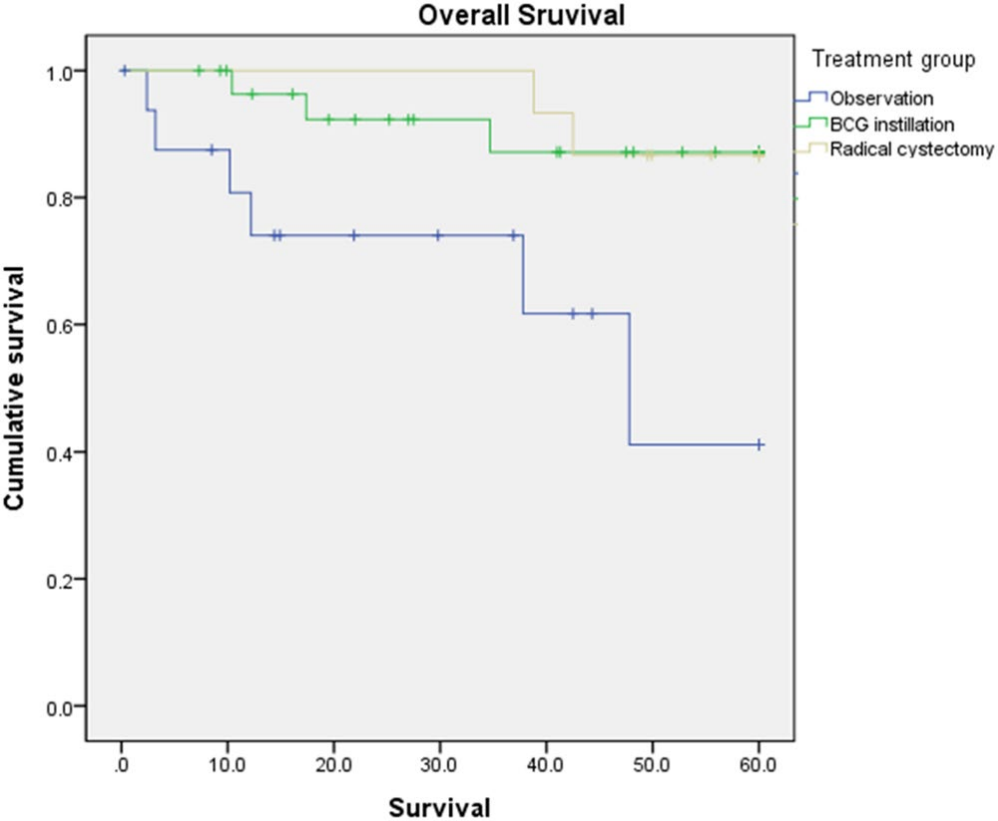
Kaplan-Meier curve for five-year overall survival in each of the treatment groups (observation, BCG instillation and radical cystectomy). Compared with observation alone, both treatment groups (BCG instillation and radical cystectomy) showed survival gain, and no statistically significant differences in survival gain were found with BCG instillation and radical cystectomy.

Eleven patients (17.7%) died within 5 years of treatment follow-up. The five-year OS was not significantly different between BCG instillation and radical cystectomy (p = 0.893); however, both treatments (BCG instillation and radical cystectomy) showed survival improvement compared with the observation group (p = 0.019, p = 0.013 for each) (Fig. 2). The mean survival times were 41.03 (95% CIs: 21.48–52.59), 55.16 (95% CIs: 59.95–60.38), and 57.42 (95% CIs: 54.07–60.77) months following observation, BCG instillation and radical cystectomy, respectively.

**Figure 3:**
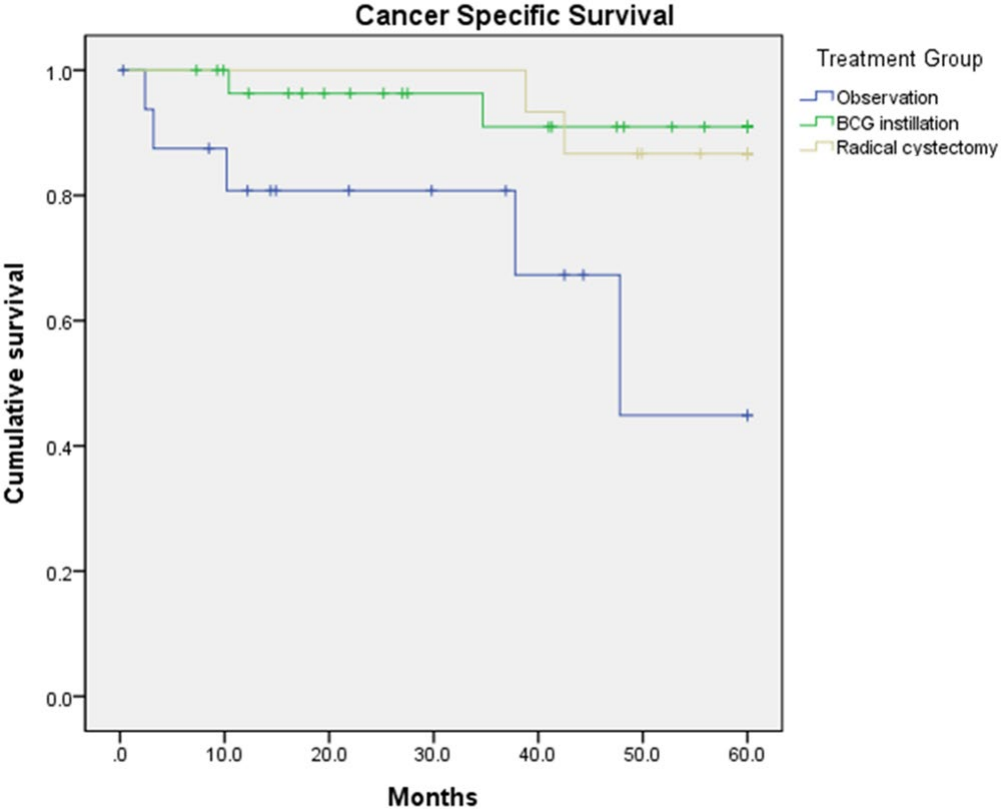
Kaplan-Meier curve for five-year cancer-specific survival in each of the treatment groups (observation, BCG instillation and radical cystectomy). Both treatment groups (BCG instillation and radical cystectomy) showed survival gain compared with observation alone, without any statistically significant differences in survival gain with BCG instillation and radical cystectomy.

The five-year cancer-specific survival also showed a statistical survival benefit following BCG instillation (p = 0.038) and radical cystectomy (p = 0.013) compared with observation alone. No statistically significant difference existed between BCG instillation and radical cystectomy (p = 0.811) (Fig. 3). The mean survival times were 42.77 (95% CIs: 37.59–54.96), 56.81 (95% CIs:52.52–61.10), and 57.42 (95% CIs:54.07–60.77) months with observation, BCG instillation and radical cystectomy, respectively.

## Discussion

The treatment of high-risk NMIBC represents a clinical grey zone. Owing to its higher chance of recurrence and progression, high-risk NMIBC warrants aggressive treatment. Current standards of treatment recommend BCG instillation for high-risk NMIBC. Specific conditions including BCG refractory or aggressive histologic variants require early radical cystectomy. Although squamous or glandular variants are the most common subtypes, no clear evidence supports the efficacy of BCG instillation in these variants. Our data suggest that both BCG instillation and radical cystectomy show similar survival gain in OS and CSS compared with observation alone in high-risk NMIBC with squamous or glandular differentiation. To the best of our knowledge, this study represents the largest and most recent evidence supporting the clinical efficacy of BCG instillation for high-risk NMIBC patients manifesting squamous or glandular subtypes.

Histological variants are generally considered higher risk than pure urothelial carcinoma^6^, warranting more aggressive treatment. However, minimal evidence is available to suggest additional treatment for histologic variants in this population subclass. The M.D. Anderson study group reported early^11^ and follow-up data^14^ of micropapillary variants with urothelial carcinoma showing a poor response to BCG instillation therapy, prompting early radical cystectomy. Plasmacytoid variants are rare in NMIBC, and associated with an 80% risk of pathologic upstaging after radical cystectomy, therefore, conservative management is not recommended^15^.

Previous studies investigated the role of BCG instillation in histologic variants of heterogenous sub-groups of patients. Shapur *et al*.^16^ investigated BCG instillation in high-risk NMIBC associated with an heterogenous histologic variant. Among the 22 patients in the target population, 11 squamous or glandular, 6 nested, 4 micropapillary and one sarcomatoid variants were compared with 144 cases of pure urothelial carcinoma. The author concluded that the five-year recurrence-free survival (RFS) and CSS were similar in both groups; however, the progression-free survival (PFS) was statistically poor in the histological variants (p = 0.02). Another study investigated the effect of BCG instillation by Gofrit *et al*.^17^ among 41 high-risk NMIBC patients with histologic variants including 14 micropapillary, 13 squamous, 9 glandular and 7 nested variants, compared with 140 cases of pure urothelial carcinoma. In this study, all the five-year RFS, PFS, OS and CSS were worse in the histologic variants. Squamous or glandular differentiation variants are the most common subtypes; however, no clinical evidence of intravesical BCG instillation is available^10^. A recent study from Japan^18^ explored the clinical significance of BCG instillation therapy only in squamous or glandular differentiation using the data from 41 NMIBC patients. Based on a comparison of 20 BCG-treated and 21 non-BCG treated patients (included 15 observations), the study concluded that BCG instillation had a positive effect on PFS and CSS. Our results were similar to those reported by Yorozuwa *et al*.^18^ using 62 squamous or glandular subtypes; however, a more selective cohort of high-risk NMIBC revealed the clinical benefit of BCG instillation in terms of OS and CSS.

Histological variants of NMIBC are associated with the risk of under staging. Weizer *et al*.^19^ reported that mixed histological variants increased the risk of under staging, which may adversely impact survival. Kamat *et al*.^11^ reported the pathological upstaging of 42% of micropapillary variants after early radical cystectomy. Squamous or glandular differentiation was associated with higher stages of disease and pathologic upstaging^8,20^, warranting radical cystectomy in some patients. Our data suggest that radical cystectomy resulted in a clear survival benefit for 15 patients in terms of both OS (p = 0.013) and CSS (p = 0.013) compared with the observation group. All the cystectomy cases included clinical T1 involving TURBT specimen. OS (p = 0.893) and CSS (p = 0.811) were not substantially different between BCG instillation and radical cystectomy. Therefore, we suggest that BCG instillation was an appropriate treatment option for high-risk NMIBC cases manifesting squamous or glandular histologic variants.

Our study has several limitations. First, this was designed retrospectively, and therefore, we cannot exclude the risk of bias. The average prevalence of histologic variants ranged from 10% to 25% in studies; however, only 8.8% of patients showed histological variants in our study, which may suggest a potential risk of selection bias. To overcome these limitations, we selected homogenous disease groups of high-risk NMIBC. However, this attempt provided only limited number of eligible patients for this study. The small sample size of 62 target patients analyzed in this study is another limitation. The selection of treatment modality followed by surgeon’s and patient’s preference, and this should be affected on lower survival of observation group. Despite these limitations, this study included the largest number of eligible patients and successfully demonstrated the clinical impact of BCG instillation therapy compared with all the eligible treatment options. This result highlights the role of effective treatment strategies for high-risk NMIBC with squamous or glandular variants. Further large-scale, prospective studies are needed to establish the efficacy of different treatment strategies unequivocally.

## Conclusion

In high-risk NMIBC bladder cancer with squamous or glandular histological variants, treatment with intravesical BCG and radical cystectomy shows survival gain. No differences in OS and CSS were found with intra-vesical BCG instillation and radical cystectomy. In high-risk NMIBC with histological variants, BCG instillation represents an appropriate treatment option.
